# How COVID-19 has changed medical research funding

**DOI:** 10.1098/rsfs.2021.0025

**Published:** 2021-10-12

**Authors:** Patrick F. Chinnery, Jonathan J. Pearce, Anna M. Kinsey, Joanna M. Jenkinson, Glenn Wells, Fiona M. Watt

**Affiliations:** Medical Research Council, 58 Victoria Embankment, London E4Y 0DS, UK

**Keywords:** research, funding, COVID-19, medical

## Abstract

Here, we consider how the lessons we learned in 2020 from funding COVID-19 research could have a long-term impact on the way that we fund medical research. We look back at how UK government funding for COVID-19 medical research evolved, beginning with the early calls for proposals in February that pump-primed funding for vaccines and therapeutics, and culminating in the launch of the government's National Core Studies programme in October. We discuss how the research community mobilized to submit and review grants more rapidly than ever before, against a background of laboratory and office closures. We also highlight the challenges of running clinical trials as the number of hospitalized patients fluctuated with different waves of the disease.

## 1. Background

The Medical Research Council (MRC) was founded in 1913, as the Medical Research Committee and Advisory Council, in order to distribute UK government funding for medical research under the terms of the National Insurance Act (1911). This Act provided health and unemployment insurance for workers and specified free institutional treatment for tuberculosis. While the MRC was born out of a great pandemic—one that is still raging, leading to 1.4 million deaths in 2019—it has subsequently had far-reaching effects on medical research, an early illustration of how pandemics accelerate change.

Now part of UK Research and Innovation (UKRI), MRC's mission is to improve human health through world-class research. MRC funds a wide portfolio of research, from blue-skies discovery science to clinical trials and research into disease prevention. MRC was therefore an obvious choice of organization, leadership and funding to help support COVID-19 research when the pandemic hit in 2020.

In fact, MRC's association with coronavirus research dates back to the discovery of this class of virus. The first human coronaviruses were isolated by researchers working at the MRC's Common Cold Unit [1]. The Unit, which was open from 1946 until 1989, was distinctive because it conducted both laboratory-based experiments and research on human volunteers. The common cold can be caused by a large number of different viruses and, among them, Almeida and Tyrell [2] described strains that had particles shaped like a crown when viewed by electron microscopy. Early work on the effects of coronaviruses on cultured epithelial cells presaged the damage that severe acute respiratory syndrome (SARS)-CoV2-2, the coronavirus responsible for COVID-19, does to the lung and other tissues [3].

While the multiplicity of viruses that cause the common cold and the mild nature of the illness led researchers to abandon the search for a cure, two more dangerous coronaviruses—those that cause SARS and Middle East respiratory syndrome (MERS)—emerged this century. In addition, there has been a growing appreciation that global changes in land use are creating more hazardous interfaces between humans and the reservoirs of zoonotic diseases found in livestock and wildlife [4]. These considerations alerted researchers to the need to prepare for future epidemics. The resulting initiatives, which have been important in tackling COVID-19, include the International Severe Acute Respiratory and Emerging Infection Consortium (ISARIC), which is a global federation of clinical research networks (CRNs), and the UK Vaccine Network, a partnership between the Department of Health and Social Care (DHSC), MRC and the Biotechnology and Bioscience Research Council. Coalition for Epidemic Preparedness Innovations (CEPI), which was founded in Davos in 2016 by the Norwegian and Indian governments, the Gates Foundation, the Wellcome Trust and the World Economic Forum, has been of considerable importance, as has the World Health Organization (WHO) Global Coordinating Mechanism for R&D in Epidemics and the Global Research Collaboration for Infectious Disease Preparedness (GloPID-R), founded in 2013.

During 2020, other parts of the research landscape were also mobilized to tackle COVID-19. These include MRC core-funded research centres and institutes with a range of different missions—virology, protein structure, disease modelling, cell biology, population cohorts and immunology. We are fortunate in the UK to have a variety of different sources of life science research funding—from government departments and arms-length bodies to non-profit organizations (charities), such as the Wellcome Trust, large pharmaceutical companies and small biotech start-ups. A further asset is that the government launched a Life Sciences Industrial Strategy in 2017, which has had the effect of bringing together senior individuals across government, funders, regulators, pharma and biotech on a regular basis, fostering good cross-sector communication. All played an important part in the COVID-19 pandemic.

## 2. Early reports of the new disease

Anna Kinsey, MRC's virology lead, was tracking the emergence of COVID-19 from early January through our membership of GloPID-R and the WHO Global Coordinating Mechanism for R&D in Epidemics. Our direct involvement in funding COVID-19 research was stimulated by the data from GloPID-R and WHO and inquiries from reseachers, including an e-mail to the MRC Executive Chair (FMW) from Robin Shattock, Head of Mucosal Infection and Immunity in the Department of Medicine at Imperial College London. The title of his e-mail was ‘Rapid vaccine response to Wuhan Coronavirus' and it was sent on 23 January 2020. In it, Professor Shattock expressed his belief that his team could rapidly develop a vaccine for the new virus, but he needed funding. It was clear from his e-mail that MRC was not Professor Shattock's first port of call for funding, which ended ‘I think this is an exceptional circumstance that might require some creative decision making’.

FMW forwarded Professor Shattock's e-mail to Chris Whitty (Chief Medical Officer (CMO) for England & Chief Medical Advisor to the UK Government and head of the National Institute for Health Research, NIHR) and Jeremy Farrar (head of the Wellcome Trust). This led to a conference call on 27 January with Chris Whitty, Jeremy Farrar, Mark Walport (then head of UKRI) and Patrick Vallance (UK government Chief Scientific Advisor). We agreed to run a rapid open joint call for proposals—vaccines were seen as one of the top priorities. On a subsequent call between funders, on 28 January, Anna and Joanna Jenkinson (JJ), our Head of Infections and Immunity, argued successfully for the inclusion of therapeutics and all the other areas in the draft WHO R&D blueprint. MRC also put additional funds into several core investments, including Neil Ferguson's MRC Centre for Global Infectious Disease Analysis (GIDA), the MRC Centre for Virus Research in Glasgow and the MRC Protein Phosphorylation and Ubiquitylation Unit in Dundee. We made supplementary awards to the MRC Units in Gambia and Uganda, who worked closely with their governments to roll out COVID-19 vaccines.

Jonathan Pearce (JP), Director of the MRC Covid-19 response, was key to launching the new funding call because he previously developed an MRC rapid response mechanism building on experience of the Ebola rapid response and further developed through the Zika rapid response. He responded to FMW's e-mail request for advice: ‘While different funders may look to support different elements of the proposed research, having a shared process could increase efficiency, as many of the relevant experts will be able to contribute to multiple agendas, improve portfolio balance and oversight, and send a clearer message to community’. In an e-mail on 28 January, JP quoted from a 2015 MRC funding call at the time of the Zika virus outbreak: ‘Short term (12–18 month) proposals are sought that will provide novel, critical and timely insights into the nature of the risk posed by the Zika virus and/or potential avenues for its management or prevention’. He suggested a similar approach for COVID-19.

## 3. A multi-faceted approach to COVID-19 research

**Figure 1.**
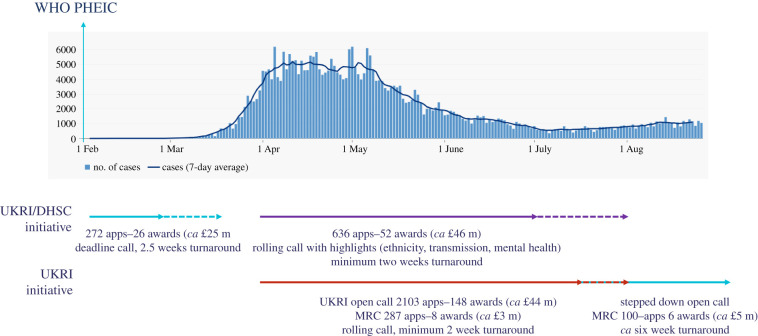
Timeline of COVID-19 funding calls with MRC involvement. UK cases shown.

As a result of the discussions at the end of January, the UKRI/DHSC COVID-19 rapid response initiative was launched on 4 February 2020, with an initial budget of £20 million. MRC and NIHR provided the secretariat led by JJ and Dr Mike Rogers (NIHR) (figure 1). The timing of the announcement was significant. On Friday 31 January, Jeremy Farrar tweeted a pledge from Wellcome of up to £10 million to accelerate research to support global efforts to tackle the coronavirus epidemic, highlighting an ongoing initiative on Research in Epidemic Preparedness and Response in collaboration with the Foreign, Commonwealth and Development Office (FCDO). However, we were instructed to keep quiet until 4 February because 31 January was ‘Brexit Day’ and the call could be interpreted as a distraction.

The first UKRI/DHSC call, with a focus on vaccines and therapeutics, closed on 13 February and received 58 applications that were assessed on 2 March. At this stage, schools were still open and so the closing date for the second call was set as 27 February, to avoid the half-term holiday the week before. It also enabled applicants and the call's panels to reflect on the COVID-19 research priorities identified at a WHO and GLoPID-R Global Innovation Forum on 11 and 12 February. The call panels met on 17 March to consider the 213 applications received. The remit of the second call was diagnostics, clinical, epidemiology, anthropology, social sciences and other types of underpinning research.

The inclusion of anthropology and social sciences, with the support of colleagues from the Economic and Social Research Council (ESRC), was the first time that these disciplines had been included in an emergency call and reflected growing recognition of the need for an interdisciplinary approach to pandemic preparedness and response. An example of the value of the inclusion of these wider perspectives is the CoMix study supported by the call (Professor John Edmunds, LSHTM). CoMix was designed as a social contact survey, collecting data on epidemiologically relevant social interactions from two panels of 2500 individuals. The data provided direct insights into contact patterns in the community and were used to estimate changes in the reproduction number some weeks ahead of epidemiological data, enabling the impact of non-pharmaceutical interventions to be inferred [5].

In addition to its speed, the calls we ran in February and March, which supported 26 projects (a total of approximately £25 million), had several unusual features [6]. The amount of money that researchers could apply for was not specified; we asked applicants simply to let us know how much money they needed. The calls were open to small and large companies, and NIHR funding for NHS Trusts was provided across the whole of the UK. International collaborations were encouraged, including with China. In the first round, applications ranged from under £50 000 to over £7 million, a much larger spread than we would have anticipated. Another feature, harking back to the Zika call, was that proposals had to show how the research could make a valuable contribution to the understanding, diagnosis, prevention and/or management of the COVID-19 outbreak within 18 months; normally, we would fund projects for 3–5 years. In addition, although applications underwent independent peer review, as is our normal practice, the final funding decision lay with Chris Whitty, as CMO, thereby ensuring immediate policy linkage to bodies such as NERVTAG.

Finally, data and tools/reagents generated under the call had to be made widely available with immediate effect. While this is true of all of the research we fund, the speed of sharing could not be dictated by the normal publication process and indeed preprint servers such as bioRxiv and medRxiv really came into their own during the pandemic. In quoting findings from preprints, scientists and journalists were careful to add a caveat when work had yet to be peer-reviewed. For a variety of reasons, some preprints never progress to publication and time will tell whether or not the conversion rate is lower for COVID-19 preprints. A striking illustration of the need to rely on preprints rather than waiting for peer-reviewed publication is that the antihelminthic drug niclosamide was being evaluated as a potential treatment a year before some of the definitive supporting evidence [7] was published.

There were so many applications to consider at the 17 March meeting that we had to run three panels concurrently, and by that date, people were starting to avoid travel. Approximately one-third of the panelists participated remotely and from March onwards all MRC panels met via Zoom. The extraordinary response from our community in agreeing to sit on the COVID panels at very short notice was remarkable. In addition to being leaders in their research fields, many had mounting clinical responsibilities and were tackling the considerable logistical challenges of preparing their hospitals and universities for the impending lockdown. Jonathan Van Tam, the deputy CMO, briefed the panelists and answered questions about how fast the disease was spreading—the questions were as much personal curiosity regarding the pandemic as about the funding priorities. Travelling through the streets of central London that day was an eerie and sad experience—the hand-written signs on the doors of shops and restaurants were a harbinger of the lockdown that began on 23 March.

## 4. Need for speed

We realized that although the two calls worked well the disease was moving fast and we had to establish a more agile approach [8]. This resulted in a DHSC/UKRI rolling call (figure 1), building on the first two calls and specifying impact on an even more aggressive time frame: 12 months. This rolling call, which received *ca* 650 applications in the period it was open (end of March to end of June), and made 52 awards (total value approximately £46 million) had a target interval from submission to decision of 10 days. This placed considerable strain on our secretariat, which continued to be led by JJ and Mike Rogers, many of whom were working from home while managing caring responsibilities and keeping on top of their other NIHR and MRC duties.

The rolling call engaged the entire research community. There was representation on the panels, which met weekly, from across England and the devolved nations, and the call was able to respond to shifting priorities as the pandemic evolved. The expert reviewers worked extremely hard and enjoyed the camaraderie and opportunity to carry out work of central importance to the UK and internationally, although some fatigue and dissatisfaction inevitably crept in. It is hard to make informed decisions about a completely new disease as the available data emerge, and are sometimes contradicted, daily. To support coordination of cross government efforts, the research priorities of the rolling call were informed by the UK government's Scientific Advisory Group for Emergencies (SAGE) priorities. The priorities of the cross governmental Diagnostics, Vaccines and Therapeutics Taskforces also informed decision making. MRC participated in these, with Glenn Wells (GW), newly appointed MRC Director of Strategy & Planning, playing an important role. As an illustration of how quickly everyone was being pressed into service, GW joined MRC on 1 April and was seconded to his Taskforce role on 13 April, Easter Sunday.

We worked hard to assemble the right teams of researchers at the right scale to tackle some of the major challenges thrown up by the disease, whether in immunology, obesity, mental health or the disproportionate toll of COVID-19 on Black, Asian and Minority Ethnic (BAME) communities. ISARIC-4C, the world's largest study of COVID-19 hospitalized patients, identified key risk factors of age, sex, obesity and ethnicity, and we held a highlighted call with DHSC to better understand the unequal burden of the disease on our BAME populations, since there had been a lack of applications to the open call on this important issue. The same rationale applied to issuing transmission and mental health highlight notices.

We worked closely with the community, including through targeted nested highlight calls, to address the issues being thrown up by the disease. To some extent we could be seen as moving into ‘contract research’ and there was a danger that while we were trying to corral the major luminaries in a field to come together, there were more junior, unknown, researchers who were better placed to do the heavy lifting. We noticed a tendency to attract proposals that overly reflected the applicants' narrow starting point of interest rather than being needs driven.

The pandemic was obviously an international health emergency and several efforts aimed at collating and sharing information internationally were important. One example was the GloPID-R-UKCDR COVID-19 Research Tracker (https://www.ukcdr.org.uk/covid-circle/), to which all MRC awards were added, thereby improving community understanding of the funding portfolio. Another is an analysis of the effect of frailty on patient survival, which was able to gain conclusive insights through data gathering across Europe [9].

We found that it could be hard to get the research community to focus on rapid delivery research that could truly produce a public health impact in 12 months. We also noted that the streamlined application process that was introduced specifically for the COVID calls may have encouraged weaker submissions, and over the course of the rolling call we only funded 10% of submissions, even though at that time the budget was not constrained (figure 1). There was undoubtedly a tendency of some researchers to try to shoe-horn their existing research into the rapid calls, with the aim of winning rapid funding, and this probably contributed to the low success rate. Ongoing evaluation of the outputs and impacts of the grants we funded will provide useful insights into the cumulative pros and cons of adjusting the funding clock. This will inform our funding patterns in a non-pandemic, non-rapid response mode scenario.

Running in parallel with the UKRI/DHSC initiative and in recognition that the impacts of the pandemic would extend well beyond primary health into areas including the economy, education, culture and wellbeing, UKRI launched an open call in March, in which applications were relayed to individual Councils and Innovate UK for evaluation (figure 1). This had an initial budget of £50 million, which was extended by an additional £120 million in August and made approximately 400 awards. Dr Jessica Boname led the MRC's contribution to the UKRI call, liaising closely with other Research Councils within UKRI. Again, the ease of application and speed of decision making encouraged a large number of applications that were not always of the highest quality, which was reflected in the success rate (figure 1).

The umbrella of the UKRI COVID-19 funding call enabled the academic community to apply for a diverse set of projects ranging from medical, biological to physical sciences/engineering research (https://www.ukri.org/find-covid-19-research-and-innovation-supported-by-ukri/). Topic maps provided on the website were developed to enable applicants to see priority areas and what projects had already been funded. Specific panels were set up to deal with applications. Thus, the initiative was truly cross-Council and cross-disciplinary, with strong links to funding for companies via innovation grants.

The early months of 2020 saw several innovative approaches to data gathering and data sharing that turned out to be highly beneficial to researchers and the population at large. One notable example is the COVID Symptom Tracker, a smartphone app that enabled individuals to self-report symptoms. Between 24 March, when the app became available for download in the UK, and 21 April 2 450 569 people in the UK and 168 293 in the USA had used the app to report symptoms, which was mapped to their location [10]. Tim Spector, a researcher at King's College London, used his start-up company ZOE Global Limited to build the Covid Symptom Tracker. The data collected via the app have been very informative for epidemiological studies and have shown the willingness of citizens to share their data altruistically.

## 5. Benefits of long-term investments in medical research

Complementing the new funding calls, some of our long-standing investments were crucially important. Researchers with core funding were able to redirect resources very rapidly to address specific challenges. This included the work of the MRC Centre for GIDA, led by Neil Ferguson, which was at the forefront of delivering timely analysis to inform policy responses and was influential in supporting the work of the WHO and in determining the government's approach to lockdown. The MRC Biostatistics Unit pivoted to regularly nowcasting and forecasting COVID-19 infections and deaths, with this information feeding directly into a SAGE sub-group called the Scientific Pandemic Influenza sub-group on Modelling (SPI-M) and to regional Public Health England (PHE) teams, while also supporting the work of the WHO. Abdel Babiker of the MRC Clinical Trials Unit at UCL co-led an international study showing some beneficial effects of the drug remdesivir, and we were taken aback to hear the results announced at a White House briefing. The MRC Centre for Virus Research at the University of Glasgow completed genomic sequencing and analysis of Scotland's first confirmed COVID-19 case within 48 h. The MRC Human Immunology Unit at the University of Oxford began investigating the immune response and immunopathology of the virus. The MRC/UVRI and LSHTM Research Unit Uganda carried out whole-genome sequencing of SARS-CoV-2 and began investigating the psychosocial impact of COVID-19 in Uganda. As of December 2020, the MRC Unit in the Gambia ranked 6th in the world for the percentage of patients whose SARS-CoV-2 had been sequenced and was also funded to conduct a clinical trial of potential therapeutic interventions.

We funded the COVID-19 Genomics UK (COG-UK) consortium to deliver large-scale and rapid whole-genome virus sequencing, using the new funding to bring together existing agencies. COG-UK was a partnership of NHS organizations, the UK Public Health Agencies, the Wellcome Sanger Institute and academic partners. COG-UK was supported by £20 million funding from DHSC, UKRI and the Wellcome Sanger Institute. In addition, Genomics England, in partnership with the GenOMICC consortium, began working with the NHS to deliver whole-genome sequencing of up to 20 000 COVID-19 intensive care patients, and up to 15 000 people with mild symptoms. The investment in viral genomics provided information about routes of viral transmission into and within the UK and about the appearance and spread of viral mutations [11,12]. Research into host genomics identified robust genetic signals relating to key host antiviral defence mechanisms, and mediators of inflammatory organ damage in COVID-19 [13].

The re-purposing of existing funding extended to researchers who, prior to the pandemic, had been in involved in more distantly related research activity. Examples include analysis of the immune response to COVID-19 by single-cell RNA sequencing, through the Human Cell Atlas, and a cross-funder collaboration to share reagents for diagnostics development, the COVID-19 Protein Portal. The MRC Protein Phosphorylation and Ubiquitylation Unit at the University of Dundee, working in partnership with the CVR, rapidly identified 38 proteins produced by SARS-CoV-2. Health Data Research UK produced a health data priority question set for SAGE and was awarded Gates Foundation funding to establish an international data sharing platform. The MRC Laboratory for Molecular Biology (LMB) used high-resolution microscopy and structural biology to examine how the virus enters and replicates in cells. The Francis Crick Institute studied the underpinning biology of the virus, the production of recombinant proteins from the virus, immunological responses and chemical inhibitor screening. Perhaps most remarkably, the Crick became a COVID testing centre for University College London Hospital and care homes in north London, thereby giving scientists the opportunity to serve their community in new ways and exposing them to the challenges and satisfactions of working at the clinical interface.

## 6. Vaccines

Two new vaccines were being evaluated in volunteers by summer 2020, one of which, developed in Oxford in partnership with AstraZeneca, received Medicines and Healthcare products Regulatory Agency (MHRA) approval for vaccinating patients in December. The multiplier effect of our vaccine investments—where an initial grant of approximately £2 million leveraged pharma investment 100-fold—was truly remarkable.

The reason why the Oxford vaccine ChAdOx1 nCoV19 was developed so rapidly, under Sarah Gilbert's leadership, was that it was built on the researchers' experience of developing an adenovirus-based vaccine against the MERS coronavirus with support from the UK Vaccines Network [14]. The MERS vaccine had already been tested in clinical trials and so was known to be safe and able to provoke efficient immune responses. The COVID-19 vaccine work was supported by £2.6 million through the UKRI/NIHR rapid response grants in March, which provided funding to conduct pre-clinical investigations and a phase I/II trial, and scale up the vaccine to 1 million doses by summer 2020. Sarah Gilbert was co-director of the Future Vaccine Manufacturing Research Hub (Vax-Hub), supported by the Engineering and Physical Sciences Research Council, and this was also critical for moving the vaccine towards the clinic. AstraZeneca shouldered much of the large-scale manufacturing burden. Thus to make an impact in developing novel vaccines, it was important to have already prepared for an epidemic, and to have in place funded large-scale facilities and consortia of researchers working in active collaborations.

## 7. Clinical trials

As new vaccines were developed and existing drugs identified for re-purposing, the UK regulatory bodies, the Health Research Authority (HRA) and the MHRA, moved swiftly to assess and approve applications. This speeded up approval for clinical trials (which can take months, and sometimes years) to a matter of days from the initial decision to trial a drug to the approval of the first dosing in a patient. The Secretary of State for Health and Social Care issued a Notice under the Control of Patient Information Regulations requiring NHS Digital to share confidential patient information with organizations entitled to process this for COVID-19 purposes. Thus access to GP records of UK Biobank participants was unlocked. These changes, if sustained, have the potential to transform the landscape for medical research not only for COVID-19 but also for all future medical interventions.

**Figure 2.**
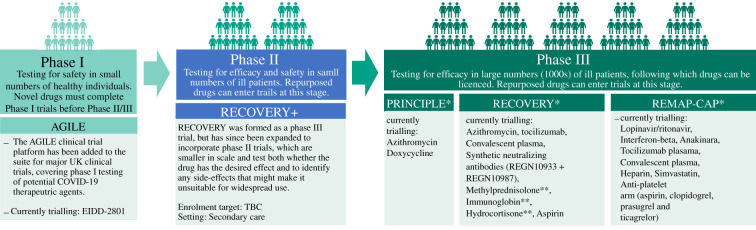
Therapeutics development pipeline. Source: DHSC Therapeutics Taskforce.

As the NHS gained experience of the severe complications of COVID-19, including dangerous inflammatory and thrombotic responses seen in some patients, the UK clinical community set out to determine whether already licenced drugs could be rapidly repurposed to reduce mortality. Multiple Phase II studies, including platform studies operating across the UK, were proposed and funded by several charities including LifeArc, and by NIHR/UKRI (figure 2). These were set up at pace, made possible by the long-standing support for clinical academia in the UK. There were notable successes. For example, during the first wave of infection the RECOVERY trial, a Phase III study, supported by the initial UKRI/DHSC call, showed the beneficial effect of dexamethasone [15], which potentially saved 1 million lives worldwide by March 2021 (https://www.england.nhs.uk/2021/03/covid-treatment-developed-in-the-nhs-saves-a-million-lives/).

In the first wave of COVID-19, RECOVERY was recruiting an average of 14% of hospitalized patients and in some NHS Trusts enrolment reached 40%. This was greatly facilitated by the NIHR CRN, which coordinates and supports high-quality research in England, and has been central to supporting a wide portfolio of clinical research studies regardless of how individual studies are funded. However, the Phase II programme in particular created a complex landscape with publicly funded studies being managed centrally alongside multiple, often competing, studies supported by commercial and charity funders.

Without national coordination and management, drugs with a similar mechanism of action were being proposed in parallel, and investigators were often unaware of the duplicated effort. Falling patient numbers after the first wave of infection made the situation worse, as the various trials competed to recruit, with none approaching closure.

**Figure 3.**
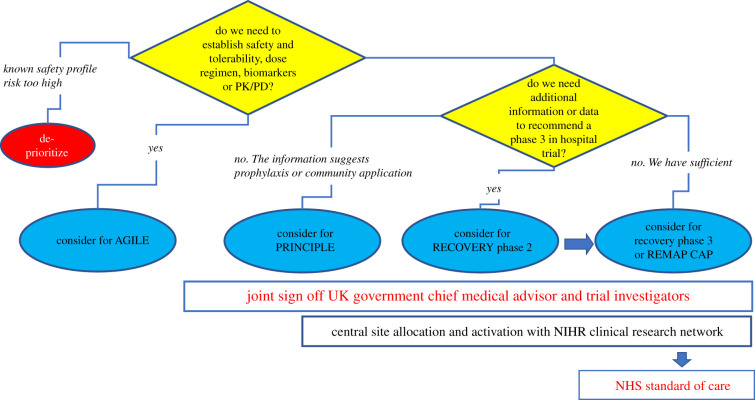
UK-CTAP logic model.

To address these issues, Patrick Chinnery, MRC Clinical Director, worked with NIHR and the CRN to build a national collaboration for the Phase II studies, in partnership with the successful RECOVERY Phase II trial platform (renamed RECOVERY+) and the DHSC Therapeutics Task Force. A key step was establishing an independent COVID-19 Therapeutics Advisory Panel (UK-CTAP) to advise the CMO on which drugs to test in Phase II and Phase III studies (figure 3). This immediately removed any duplication and ensured a balanced portfolio of drugs with complementary mechanisms of action targeting the disease mechanisms that were only just being discovered. Initially set up to ‘feed’ the clinical trials for severe COVID-19 in a hospital setting, it made sense to broaden the remit to encompass Phase I studies (AGILE), other nationally sponsored clinical trials in the intensive care setting (REMAP-CAP), and early infection in the community (PRINCIPLE), also supported by UKRI/DHSC (figure 2). As of November 2020, UK-CTAP had evaluated 128 potential drugs.

Established prior to the outbreak of COVID-19, REMAP-CAP was an international clinical trial spanning—as of November 2020—275 sites in 19 countries, testing a range of supportive care and specific interventions in patients admitted to intensive care units (ICU) with severe community-acquired pneumonia (CAP). A new arm specifically for COVID-19 was added to the trial in order to test potential COVID-19 treatments in critically ill patients in hospital settings, including ICU. As of November 2020, approximately 20% of patients in ICU within the UK were enrolled in REMAP-CAP.

RECOVERY+ tested potential COVID-19 treatments in hospital settings in patients with moderate to severe COVID-19. Given the success of the Phase III RECOVERY platform in delivering a single-platform trial across the NHS, the UK government increased investment in an expanded platform to operate for a further 24 months. This included new treatments tested in Phase II and Phase III studies being delivered through the RECOVERY+ platform in patients admitted to the hospital.

PRINCIPLE was testing potential COVID-19 treatments in the community in patients with mild to moderate COVID-19 who were aged over 65; or aged 50 to 64 with an underlying health condition. Recruitment was initially low because of low levels of GP attendance and COVID-19 patients presenting through other routes, such as calls to the emergency services and NHS Test and Trace. In addition, there were major logistical challenges in issuing swabs and sending individual packages of drugs to people at home who were self-isolating. However, patients were given the opportunity to enrol online and as of November 2020, an average of 25 patients per day was being recruited. The DHSC also supported Phase I COVID-19 therapeutic candidates through an additional Phase I national platform clinical trial, AGILE.

While the vaccine and therapeutic developments offered great promise for managing the pandemic and mitigating infection, as 2020 progressed there was a growing recognition of the lasting impacts that infection could have in both mild and severe cases. Through the UKRI/DHSC rolling call, we therefore supported PHOSP-COVID to study and address the long-term impacts on hospitalized patients and held a joint call with DHSC focused on long-COVID in community cases.

## 8. National core studies

On 28 October 2020, Patrick Vallance announced the commencement of the National Core Studies to tackle the next phase of the COVID-19 pandemic. He set out six areas (box 1) with well-defined objectives for the first six months of operation, ranging from epidemiology and surveillance through viral transmission and the long-term impacts of COVID-19 on mental and physical health. All of these had grown out of the early funding efforts by UKRI, NIHR and others, and the platforms, expertise and collaborations that had formed as a result. JP led on the delivery of three of the study areas on behalf of UKRI. In addition to their immediate impacts, the NCS has the potential to provide legacy value, as the breadth of data being connected and used, which spans health and administrative data, and the collaborations being catalysed have application well beyond COVID-19.

Box 1. **National Core Studies themes, October 2020. Source: https://committees.parliament.uk/publications/3400/documents/32493/default/.**

— **Epidemiology and Surveillance** led by Professor Ian Diamond (Office for National Statistics). *Collecting and analysing data to inform appropriate levels of restrictions and protection against imminent outbreaks*.

— **Clinical Trials Infrastructure** led by Professor Patrick Chinnery (MRC) and Dr Divya Chadha Manek (Vaccines Task Force/NIHR). *Building on established NIHR infrastructure (and equivalent in DAs) to accelerate delivery of large-scale COVID-19 trials for drugs and vaccines*.

— **Transmission and Environment** led by Professor Andrew Curran (Health and Safety Executive). *Understanding and mitigating transmission of the disease in workplace, transport and public places*.

— **Immunity** led by Professor Paul Moss (University of Birmingham). *Understanding immunity against COVID-19 to inform back-to-work policies*.

— **Longitudinal Health** led by Professor Nish Chaturvedi (University College London). *Understanding the impact of COVID-19 on long-term health to inform the design of mitigating policies (bringing together information from existing studies and cohorts)*.

— **Data and Connectivity** led by Professor Andrew Morris (Health Data Research UK in partnership with Office for National Statistics). *Making UK-wide health and administrative data available for linkage and accessible to catalyse COVID-19 research*.

## 9. Conclusion

In the wake of the COVID-19 pandemic, we can all reflect on what we got right and what we got wrong. We have certainly found a variety of ways to improve the medical research landscape. What we learned from the rapid funding calls was that we can accelerate or slow down the funding clock as necessary. This led to an ongoing agile funding panel, which reviewed submissions to the UKRI rolling call and was co-chaired by three chairs of existing MRC funding committees (the Infections and Immunity Board, the Population and Systems Medicine Board and the Developmental Pathway Funding Scheme) with membership drawn primarily from those committees. It has been meeting every six weeks (whereas the existing panels meet three times a year) and includes external written peer review.

The speed with which research ethics approval has been granted, and MHRA approval ‘proves the principle’ will make the UK very attractive for pharma to conduct clinical trials. The success of RECOVERY and other clinical trials in recruiting patients around the UK has provided opportunities for clinicians to contribute to the research agenda, regardless of geography and this will have lasting benefits for medical research.

We have learned the importance of clear and effective national leadership and coordination of the research, and how we can leverage decades of investment across the UK to respond to a global emergency. We have developed sustainable models such as the National Core Studies programme, preparing us for future threats.

We have seen rapid sharing of data and reagents. Preprint servers, including bioRvix and medRxiv, have played an essential role in rapid data sharing. This is all the more remarkable given that medRxiv, launched in June 2019, was less than a year old at the start of the pandemic. We have seen high-quality science journalism and scientists stepping up to the plate to discuss and explain their work. Twitter has been important, but so too have blogs and the tireless work of the Science Media Centre.

A further key legacy will be vaccines manufacturing capacity, previously a vulnerability for UK pandemic preparedness, that will be created. This provides opportunities that could enable us to tackle other priority pathogens and the slow silent pandemic of antimicrobial resistance. We have seen the benefit of the risk that was taken in funding epidemic preparedness platforms and the need to do more in ‘peacetime’ to create multidisciplinary networks to tackle existing and emerging infections that are then well placed to pivot their research effort when the next epidemic arises. Epidemics and the patterns of infectious diseases are changing due to climate change, urbanization and globalization and we must do more to be ready to prevent and contain the next potential pandemic.

Finally, and perhaps surprisingly, scientists with no research interests of direct relevance to COVID-19 have stepped in to support testing, whether by donating equipment or carrying out the tests, have volunteered to process blood samples for the COVID-vaccine trials and become trained to deliver vaccines. This could lead to a profound change in research culture, with an intermingling and mutual understanding of discovery and applied medical research.

## Data accessibility

This article has no additional data.
